# (*E*)-*N*′-(5-Bromo-2-hy­droxy­benzyl­idene)-3,5-dihy­droxy­benzohydrazide monohydrate

**DOI:** 10.1107/S1600536810027856

**Published:** 2010-07-17

**Authors:** Siti Munirah Saharin, Hamid Khaledi, Hapipah Mohd Ali

**Affiliations:** a Department of Chemistry, University of Malaya, 50603 Kuala Lumpur, Malaysia

## Abstract

The Schiff base mol­ecule in the title compound, C_14_H_11_BrN_2_O_4_·H_2_O, is almost planar with an r.m.s. deviation for the non-H atoms of 0.16 Å. In the crystal structure, the Schiff base mol­ecules and the water mol­ecules are linked together by inter­molecular N—H⋯O and O—H⋯O hydrogen bonds, leading to layers parallel to the *bc* plane. An intra­molecular O—H⋯N hydrogen bond involving the imine N atom and a hy­droxy substituent is also observed.

## Related literature

For the isotypic Cl analogue C_14_H_11_ClN_2_O_4_·H_2_O, see: Deng *et al.* (2009 ▶).
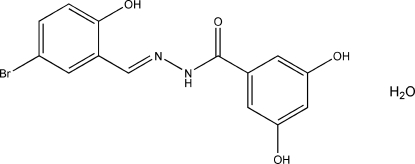


## Experimental

### 

#### Crystal data

C_14_H_11_BrN_2_O_4_·H_2_O
*M* *_r_* = 369.17
Monoclinic, 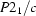

*a* = 13.5685 (3) Å
*b* = 8.0532 (2) Å
*c* = 13.2447 (2) Å
β = 100.186 (1)°
*V* = 1424.44 (5) Å^3^
*Z* = 4
Mo *K*α radiation
μ = 2.91 mm^−1^
*T* = 296 K
0.58 × 0.33 × 0.06 mm

#### Data collection

Bruker APEXII CCD diffractometer
Absorption correction: multi-scan (*SADABS*; Sheldrick, 1996 ▶) *T*_min_ = 0.283, *T*_max_ = 0.845
9148 measured reflections
2579 independent reflections
2183 reflections with *I* > 2σ(*I*)
*R*_int_ = 0.034

#### Refinement

*R*[*F* ^2^ > 2σ(*F* ^2^)] = 0.031
*wR*(*F* ^2^) = 0.077
*S* = 1.04
2579 reflections
217 parameters
6 restraints
H atoms treated by a mixture of independent and constrained refinement
Δρ_max_ = 0.45 e Å^−3^
Δρ_min_ = −0.56 e Å^−3^

### 

Data collection: *APEX2* (Bruker, 2007 ▶); cell refinement: *SAINT* (Bruker, 2007 ▶); data reduction: *SAINT*; program(s) used to solve structure: *SHELXS97* (Sheldrick, 2008 ▶); program(s) used to refine structure: *SHELXL97* (Sheldrick, 2008 ▶); molecular graphics: *X-SEED* (Barbour, 2001 ▶); software used to prepare material for publication: *SHELXL97* and *publCIF* (Westrip, 2010 ▶).

## Supplementary Material

Crystal structure: contains datablocks I, global. DOI: 10.1107/S1600536810027856/bh2299sup1.cif

Structure factors: contains datablocks I. DOI: 10.1107/S1600536810027856/bh2299Isup2.hkl

Additional supplementary materials:  crystallographic information; 3D view; checkCIF report

## Figures and Tables

**Table 1:** Hydrogen-bond geometry (Å, °)

*D*—H⋯*A*	*D*—H	H⋯*A*	*D*⋯*A*	*D*—H⋯*A*
O1—H1⋯N1	0.81 (2)	1.95 (2)	2.657 (2)	145 (3)
N2—H2*N*⋯O2	0.85 (2)	2.07 (2)	2.913 (3)	170 (2)
O11—H11⋯O8^i^	0.83 (2)	1.94 (2)	2.750 (2)	168 (3)
O13—H13⋯O1^ii^	0.79 (2)	2.19 (2)	2.959 (2)	165 (3)
O2—H2*A*⋯O8^i^	0.81 (2)	1.98 (2)	2.776 (3)	171 (4)
O2—H2*B*⋯O11^iii^	0.83 (2)	2.06 (2)	2.861 (3)	165 (3)

Symmetry codes: (i) 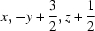; (ii) 
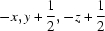
; (iii) 
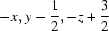
.

**Fig. 1.**
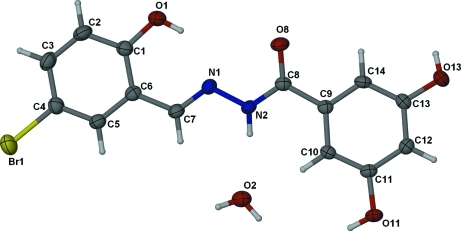
Thermal ellipsoid plot of the title compound at 50% probability level. Hydrogen atoms are drawn as spheres of arbitrary radius.

**Fig. 2.**
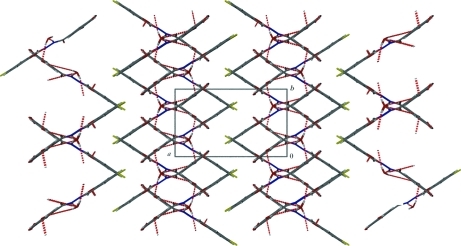
Packing view looking down the crystallographic b unit cell edge.

## supplementary crystallographic information

### Comment

The molecular structure of the title compound is shown in Fig. 1, and the crystal structure in Fig. 2. The present study shows that Br and Cl analogues (Deng *et al.*, 2009) are isotypic crystals.

### Experimental

An ethanolic solution (15 ml) of 3,5-dihydroxybenzohydrazide (0.67 g, 4 mmol) and 5-bromosalicylaldehyde (0.8 g, 4 mmol) was refluxed for 2 h. The solution was then cooled and the solid product formed was filtered off, washed with cold ethanol, and dried over silica gel. Crystals of the title compound were obtained by slow evaporation of a DMSO solution at room temperature.

### Refinement

The carbon-bound H atoms were placed in calculated positions (C—H fixed to 0.93 Å) and treated as riding on their parent carbon atoms with *U*_iso_(H) set to 1.2 *U*_eq_(carrier C). The nitrogen- and oxygen-bound H atoms were located in a difference map and refined as free atoms, with N—H and O—H distances restrained to 0.86 (2) and 0.82 (2) Å, respectively.

### Crystal data

**Table:**

C_14_H_11_BrN_2_O_4_·H_2_O	*F*(000) = 744
*M**_r_* = 369.17	*D*_x_ = 1.721 Mg m^−^^3^
Monoclinic, *P*2_1_/*c*	Mo *K*α radiation, λ = 0.71073 Å
Hall symbol: -P 2ybc	Cell parameters from 3109 reflections
*a* = 13.5685 (3) Å	θ = 3.0–26.1°
*b* = 8.0532 (2) Å	µ = 2.91 mm^−^^1^
*c* = 13.2447 (2) Å	*T* = 296 K
β = 100.186 (1)°	Plate, yellow
*V* = 1424.44 (5) Å^3^	0.58 × 0.33 × 0.06 mm
*Z* = 4	

### Data collection

**Table:**

Bruker APEXII CCD diffractometer	2579 independent reflections
Radiation source: fine-focus sealed tube	2183 reflections with *I* > 2σ(*I*)
graphite	*R*_int_ = 0.034
φ and ω scans	θ_max_ = 25.3°, θ_min_ = 3.0°
Absorption correction: multi-scan (*SADABS*; Sheldrick, 1996)	*h* = −16→16
*T*_min_ = 0.283, *T*_max_ = 0.845	*k* = −9→9
9148 measured reflections	*l* = −15→15

### Refinement

**Table:**

Refinement on *F*^2^	Primary atom site location: structure-invariant direct methods
Least-squares matrix: full	Secondary atom site location: difference Fourier map
*R*[*F*^2^ > 2σ(*F*^2^)] = 0.031	Hydrogen site location: inferred from neighbouring sites
*wR*(*F*^2^) = 0.077	H atoms treated by a mixture of independent and constrained refinement
*S* = 1.04	*w* = 1/[σ^2^(*F*_o_^2^) + (0.0328*P*)^2^ + 0.7124*P*] where *P* = (*F*_o_^2^ + 2*F*_c_^2^)/3
2579 reflections	(Δ/σ)_max_ = 0.001
217 parameters	Δρ_max_ = 0.45 e Å^−^^3^
6 restraints	Δρ_min_ = −0.56 e Å^−^^3^
0 constraints	

### Fractional atomic coordinates and isotropic or equivalent isotropic displacement parameters (Å^2^)

**Table:**

	*x*	*y*	*z*	*U*_iso_*/*U*_eq_	
Br1	0.54976 (2)	0.19522 (4)	0.67677 (2)	0.05405 (13)	
O1	0.25489 (15)	0.4914 (3)	0.33122 (13)	0.0448 (5)	
H1	0.210 (2)	0.542 (4)	0.351 (2)	0.067*	
O8	−0.01522 (14)	0.6853 (2)	0.34596 (12)	0.0375 (4)	
O11	−0.18429 (14)	0.9113 (2)	0.71231 (12)	0.0378 (4)	
H11	−0.1355 (18)	0.868 (4)	0.749 (2)	0.057*	
O13	−0.29944 (14)	1.0800 (2)	0.37286 (13)	0.0440 (5)	
H13	−0.285 (3)	1.075 (4)	0.3181 (17)	0.066*	
N1	0.15274 (14)	0.5993 (2)	0.47190 (14)	0.0279 (4)	
N2	0.07629 (15)	0.6835 (2)	0.50575 (14)	0.0277 (4)	
H2N	0.088 (2)	0.712 (3)	0.5686 (14)	0.033*	
C1	0.31939 (19)	0.4270 (3)	0.41219 (17)	0.0312 (5)	
C2	0.3994 (2)	0.3354 (3)	0.39071 (19)	0.0402 (6)	
H2	0.4072	0.3208	0.3229	0.048*	
C3	0.4676 (2)	0.2657 (3)	0.4683 (2)	0.0391 (6)	
H3	0.5211	0.2037	0.4534	0.047*	
C4	0.45524 (18)	0.2895 (3)	0.56914 (18)	0.0330 (6)	
C5	0.37678 (18)	0.3807 (3)	0.59223 (17)	0.0319 (5)	
H5	0.3701	0.3951	0.6603	0.038*	
C6	0.30662 (17)	0.4524 (3)	0.51387 (16)	0.0278 (5)	
C7	0.22369 (18)	0.5450 (3)	0.54062 (17)	0.0300 (5)	
H7	0.2219	0.5656	0.6094	0.036*	
C8	−0.00598 (18)	0.7237 (3)	0.43815 (16)	0.0266 (5)	
C9	−0.08615 (17)	0.8158 (3)	0.47789 (16)	0.0248 (5)	
C10	−0.09485 (18)	0.8153 (3)	0.58150 (16)	0.0274 (5)	
H10	−0.0499	0.7559	0.6293	0.033*	
C11	−0.17126 (17)	0.9044 (3)	0.61131 (16)	0.0276 (5)	
C12	−0.23974 (18)	0.9932 (3)	0.54147 (17)	0.0303 (5)	
H12	−0.2908	1.0530	0.5632	0.036*	
C13	−0.23079 (18)	0.9912 (3)	0.43882 (16)	0.0291 (5)	
C14	−0.15405 (17)	0.9038 (3)	0.40695 (16)	0.0283 (5)	
H14	−0.1479	0.9040	0.3381	0.034*	
O2	0.12746 (16)	0.7432 (3)	0.72574 (13)	0.0428 (5)	
H2A	0.091 (2)	0.772 (4)	0.764 (2)	0.064*	
H2B	0.153 (3)	0.656 (3)	0.750 (2)	0.064*	

### Atomic displacement parameters (Å^2^)

**Table:**

	*U*^11^	*U*^22^	*U*^33^	*U*^12^	*U*^13^	*U*^23^
Br1	0.03931 (19)	0.0753 (2)	0.04679 (19)	0.02026 (15)	0.00548 (13)	0.01280 (14)
O1	0.0449 (12)	0.0626 (13)	0.0261 (9)	0.0149 (10)	0.0044 (8)	−0.0005 (8)
O8	0.0353 (10)	0.0545 (11)	0.0224 (8)	0.0033 (9)	0.0047 (7)	−0.0065 (7)
O11	0.0417 (11)	0.0525 (11)	0.0209 (8)	0.0101 (9)	0.0098 (7)	0.0018 (7)
O13	0.0447 (11)	0.0571 (12)	0.0291 (9)	0.0212 (10)	0.0033 (8)	0.0079 (8)
N1	0.0269 (11)	0.0292 (10)	0.0281 (10)	0.0012 (9)	0.0061 (8)	−0.0039 (8)
N2	0.0273 (11)	0.0341 (11)	0.0221 (9)	0.0035 (9)	0.0048 (8)	−0.0063 (8)
C1	0.0308 (13)	0.0351 (13)	0.0275 (12)	0.0001 (11)	0.0047 (10)	0.0000 (10)
C2	0.0397 (16)	0.0533 (17)	0.0302 (13)	0.0037 (13)	0.0133 (11)	−0.0057 (11)
C3	0.0316 (15)	0.0451 (15)	0.0431 (15)	0.0054 (12)	0.0135 (11)	−0.0041 (11)
C4	0.0266 (14)	0.0382 (14)	0.0338 (13)	0.0010 (11)	0.0039 (10)	0.0021 (10)
C5	0.0316 (14)	0.0382 (13)	0.0271 (12)	0.0016 (11)	0.0082 (10)	−0.0020 (10)
C6	0.0268 (13)	0.0294 (12)	0.0276 (11)	−0.0035 (10)	0.0058 (9)	−0.0041 (9)
C7	0.0306 (13)	0.0348 (13)	0.0251 (11)	0.0009 (11)	0.0066 (10)	−0.0049 (9)
C8	0.0278 (13)	0.0289 (12)	0.0233 (12)	−0.0034 (10)	0.0053 (9)	−0.0001 (9)
C9	0.0243 (12)	0.0269 (11)	0.0233 (11)	−0.0032 (9)	0.0041 (9)	−0.0015 (9)
C10	0.0290 (13)	0.0307 (12)	0.0216 (11)	0.0007 (10)	0.0015 (9)	0.0012 (9)
C11	0.0301 (13)	0.0315 (12)	0.0222 (11)	−0.0034 (10)	0.0072 (9)	−0.0009 (9)
C12	0.0290 (13)	0.0327 (13)	0.0304 (12)	0.0029 (10)	0.0080 (10)	−0.0006 (9)
C13	0.0289 (13)	0.0299 (12)	0.0271 (11)	0.0012 (10)	0.0008 (10)	0.0032 (9)
C14	0.0322 (14)	0.0340 (13)	0.0182 (10)	−0.0018 (11)	0.0034 (9)	−0.0002 (9)
O2	0.0497 (13)	0.0487 (11)	0.0308 (10)	0.0051 (10)	0.0093 (8)	0.0000 (8)

### Geometric parameters (Å, °)

**Table:**

Br1—C4	1.899 (2)	C4—C5	1.372 (3)
O1—C1	1.361 (3)	C5—C6	1.402 (3)
O1—H1	0.812 (18)	C5—H5	0.9300
O8—C8	1.244 (3)	C6—C7	1.445 (3)
O11—C11	1.381 (3)	C7—H7	0.9300
O11—H11	0.827 (18)	C8—C9	1.487 (3)
O13—C13	1.361 (3)	C9—C14	1.389 (3)
O13—H13	0.785 (18)	C9—C10	1.398 (3)
N1—C7	1.279 (3)	C10—C11	1.375 (3)
N1—N2	1.379 (3)	C10—H10	0.9300
N2—C8	1.341 (3)	C11—C12	1.388 (3)
N2—H2N	0.851 (17)	C12—C13	1.386 (3)
C1—C2	1.383 (4)	C12—H12	0.9300
C1—C6	1.403 (3)	C13—C14	1.383 (3)
C2—C3	1.375 (4)	C14—H14	0.9300
C2—H2	0.9300	O2—H2A	0.807 (18)
C3—C4	1.389 (3)	O2—H2B	0.825 (18)
C3—H3	0.9300		
			
C1—O1—H1	110 (2)	N1—C7—C6	121.5 (2)
C11—O11—H11	109 (2)	N1—C7—H7	119.2
C13—O13—H13	108 (3)	C6—C7—H7	119.2
C7—N1—N2	116.85 (18)	O8—C8—N2	121.5 (2)
C8—N2—N1	119.13 (18)	O8—C8—C9	121.2 (2)
C8—N2—H2N	125.0 (18)	N2—C8—C9	117.29 (18)
N1—N2—H2N	115.7 (18)	C14—C9—C10	120.3 (2)
O1—C1—C2	117.4 (2)	C14—C9—C8	117.02 (19)
O1—C1—C6	122.0 (2)	C10—C9—C8	122.7 (2)
C2—C1—C6	120.6 (2)	C11—C10—C9	118.6 (2)
C3—C2—C1	120.9 (2)	C11—C10—H10	120.7
C3—C2—H2	119.6	C9—C10—H10	120.7
C1—C2—H2	119.6	C10—C11—O11	122.1 (2)
C2—C3—C4	118.8 (2)	C10—C11—C12	121.8 (2)
C2—C3—H3	120.6	O11—C11—C12	116.1 (2)
C4—C3—H3	120.6	C13—C12—C11	118.9 (2)
C5—C4—C3	121.3 (2)	C13—C12—H12	120.5
C5—C4—Br1	119.64 (18)	C11—C12—H12	120.5
C3—C4—Br1	119.10 (19)	O13—C13—C14	122.5 (2)
C4—C5—C6	120.5 (2)	O13—C13—C12	117.0 (2)
C4—C5—H5	119.8	C14—C13—C12	120.4 (2)
C6—C5—H5	119.8	C13—C14—C9	119.91 (19)
C5—C6—C1	117.9 (2)	C13—C14—H14	120.0
C5—C6—C7	119.1 (2)	C9—C14—H14	120.0
C1—C6—C7	123.0 (2)	H2A—O2—H2B	105 (3)

### Hydrogen-bond geometry (Å, °)

**Table:**

*D*—H···*A*	*D*—H	H···*A*	*D*···*A*	*D*—H···*A*
O1—H1···N1	0.81 (2)	1.95 (2)	2.657 (2)	145 (3)
N2—H2N···O2	0.85 (2)	2.07 (2)	2.913 (3)	170 (2)
O1—H1···O2^i^	0.81 (2)	2.52 (3)	2.942 (3)	114 (3)
O11—H11···O8^ii^	0.83 (2)	1.94 (2)	2.750 (2)	168 (3)
O13—H13···O1^iii^	0.79 (2)	2.19 (2)	2.959 (2)	165 (3)
O2—H2A···O8^ii^	0.81 (2)	1.98 (2)	2.776 (3)	171 (4)
O2—H2B···O11^iv^	0.83 (2)	2.06 (2)	2.861 (3)	165 (3)

Symmetry codes: (i) *x*, −*y*+3/2, *z*−1/2; (ii) *x*, −*y*+3/2, *z*+1/2; (iii) −*x*, *y*+1/2, −*z*+1/2; (iv) −*x*, *y*−1/2, −*z*+3/2.
